# Clinical Phenotypes and Genetic Findings in Very-Early-Onset Inflammatory Bowel Disease: A Vietnamese Pediatric Cohort Study

**DOI:** 10.3390/children13050666

**Published:** 2026-05-11

**Authors:** Manh Cuong Nguyen, Thi Viet Ha Nguyen, Loi Nguyen, Thuy Ha Dang, Tam Tran, Thi Van Anh Nguyen, Ngoc Thach Hoang, Ngoc Quynh Le Nguyen, Thi Minh Phuong Do, Van Tinh Nguyen, Hai Yen Vu, Thi Ngoc Hong Nguyen, Thi Thu Trang Nguyen, Thi Cam Van Le, Thi Khanh Ngoc Bui, Thi Thuy Hang Le, Minh Dien Tran

**Affiliations:** 1 Department of Pediatrics, Military Hospital 103, Vietnam Military Medical University, Hanoi 100000, Vietnam; dr.manhcuong@vmmu.edu.vn (M.C.N.); hangv103@gmail.com (T.T.H.L.); 2 Department of Pediatrics, Hanoi Medical University, Hanoi 100000, Vietnam; dominhphuong@hmu.edu.vn; 3 Department of Gastroenterology, National Children’s Hospital, Hanoi 100000, Vietnam; nguyenloins@nch.gov.vn (L.N.); thuyha@nch.gov.vn (T.H.D.); tinhnv@nch.gov.vn (V.T.N.); yenvh@nch.gov.vn (H.Y.V.); hongntn@nch.gov.vn (T.N.H.N.); thutrang@nch.gov.vn (T.T.T.N.); camvan150198@gmail.com (T.C.V.L.); 4 Department of Medicine, Washington University School of Medicine, Saint Louis, MO 63110, USA; tamt@wustl.edu; 5 Department of Immunology-Allergy-Rheumatology, National Children’s Hospital, Hanoi 100000, Vietnam; vananhnch@nch.gov.vn; 6 Department of Pathology, National Children’s Hospital, Hanoi 100000, Vietnam; hoangthach@nch.gov.vn; 7 Stem Cells Center, National Children’s Hospital, Hanoi 100000, Vietnam; quynhle@nch.gov.vn; 8 Department of Nutrition, National Children’s Hospital, Hanoi 100000, Vietnam; ngocbtk@nch.gov.vn; 9 National Children’s Hospital, Hanoi 100000, Vietnam

**Keywords:** monogenic diseases, very-early-onset inflammatory bowel disease (VEO-IBD), whole exome sequencing (WES)

## Abstract

**Highlights:**

**What are the main findings?**

- A high proportion of Vietnamese children with VEO-IBD had identifiable monogenic etiologies (~30%), predominantly involving IL10RA/IL10RB, XIAP, FOXP3, and TNFAIP3.
- VEO-IBD presented early in life with severe intestinal involvement, frequent extraintestinal manifestations, growth impairment, and aggressive disease behavior. Monogenic disease was associated with distinct genotype–phenotype correlations, including syndromic features and poor response to conventional therapies.

**What are the implications of the main findings?**

- Early integration of genomic testing is essential for children with suspected VEO-IBD, particularly those with severe, atypical, or refractory disease.
- Molecular diagnosis enables mechanism-based therapy, including targeted biologics or hematopoietic stem cell transplantation in selected patients.

**Abstract:**

**Background/Objectives:** Very early onset inflammatory bowel disease (VEO-IBD), frequently associated with monogenic defects, is increasingly recognized worldwide but remains poorly characterized in Vietnam. This study aimed to characterize the clinical phenotypes and genetic spectrum of Vietnamese children with VEO-IBD. **Methods**: We conducted a retrospective cohort study at a tertiary pediatric referral center in Vietnam from July 2016 to January 2026. Clinical, laboratory, endoscopic, histopathological, genetic, and treatment data were systematically collected and analyzed. Monogenic variants were identified using next-generation sequencing and classified according to ACMG criteria. **Results**: Thirty-six children were included, with a median age at onset of 7.5 months, and 72.2% presenting before 24 months. Crohn’s disease predominated (72.2%). Disease burden was high, with growth impairment in 75.0% and anemia in 91.7%. Extraintestinal manifestations were frequent, particularly recurrent infections (72.2%), dermatitis (44.4%), and oral ulcers (44.4%). Perianal disease occurred in 58.3%, with early complications including perianal ulcer (44.4%), perianal abscess (30.6%) and fistulas (33.3%). Inflammatory markers were markedly elevated, and disease activity indices indicated moderate-to-severe disease at diagnosis. Genetic testing was performed in 91.7% of patients, identifying monogenic etiologies in 30.3%. Identified variants involved genes related to immune regulation (IL10RA/IL10RB, FOXP3, XIAP), autoinflammation (TNFAIP3), host defense (CYBB), and epithelial function (MYO5B). **Conclusions**: Monogenic etiologies account for a substantial proportion of VEO-IBD and are associated with distinct clinical phenotypes and therapeutic implications. Early integration of genomic testing with clinical phenotyping is essential to improve diagnostic precision and enable pathway-based treatment, supporting precision medicine in pediatric IBD.

## 1. Introduction

Inflammatory bowel disease (IBD) comprises chronic inflammatory disorders of the gastrointestinal tract. It has shown a rising global incidence, including pediatric populations; however, data on very-early-onset IBD (VEO-IBD) remain limited [1]. VEO-IBD, defined as disease onset before 6 years of age, represents a distinct clinical entity characterized by atypical phenotypes, a higher prevalence of monogenic defects or inborn error of immunity (IEIs), diagnostic challenges, and variable response to conventional therapies compared with later-onset IBD [2]. Early recognition of this subgroup is important because diagnostic approaches, therapeutic strategies, and long-term outcomes may differ substantially.

Advances in genomic technologies, particularly whole-exome sequencing (WES), have transformed understanding of VEO-IBD pathogenesis [3]. A proportion of cases are now recognized as monogenic disorders involving defects in immune regulation, epithelial barrier and epithelial response defects, immunodeficiencies affecting granulocyte and phagocyte activity, and hyper and autoinflammatory disorders [4,5]. Identifying such variants may guide targeted therapies, inform prognosis, and in selected cases support curative interventions such as hematopoietic stem cell transplantation (HSCT) [2,6]. However, the reported prevalence of monogenic disease varies widely across studies, partly reflecting differences in cohort characteristics, genetic testing strategies, and geographic background [1,5]. Moreover, the clinical relevance of genetic variants identified outside classical IBD pathways, particularly those associated with immune dysregulation or metabolic disorders, remains uncertain, as they may represent primary disease drivers, modifiers of intestinal inflammation, or incidental findings, highlighting the need for integrated clinical genetic evaluation in VEO-IBD [6,7,8].

Despite growing interest in VEO-IBD, most available data originate from North America and Europe, while evidence from Southeast Asia, particularly Vietnam, remains limited [1,9]. This gap restricts understanding of regional genetic diversity, clinical phenotype variability, and treatment outcomes, particularly in settings where access to advanced genetic testing may differ [5]. Comprehensive characterization integrating clinical phenotype, laboratory markers, endoscopic findings, and genetic data is therefore essential to improve early diagnosis and individualized management.

In this context, the present study aimed to characterize the clinical phenotypes and genetic findings of children with VEO-IBD in a Vietnamese pediatric cohort. We further explored genotype–phenotype associations to better understand the potential contribution of genetic variants to VEO-IBD.

## 2. Materials and Methods

### 2.1. Study Design and Patients

This retrospective observational study was conducted at Vietnam National Children’s Hospital from July 2016 to January 2026. During the study period, a total of 114 children were diagnosed with inflammatory bowel disease (IBD), including 36 (31.57%) classified as very-early-onset IBD (VEO-IBD). Children diagnosed with VEO-IBD, defined as onset before 6 years of age, were eligible for inclusion. The diagnosis of Pediatric Inflammatory Bowel Disease (P-IBD) was established according to the revised Porto criteria of the European Society for Pediatric Gastroenterology, Hepatology and Nutrition (ESPGHAN), based on endoscopic and histopathological findings or radiographic evaluation [7]. Patients initially suspected of having IBD but subsequently identified with infectious, eosinophilic gastrointestinal, or non-inflammatory conditions were excluded from the analysis.

The primary outcome of this study was the descriptive characterization of the clinical phenotypes and genetic findings of children with VEO-IBD. Secondary outcomes included endoscopic and histopathological characteristics, treatment patterns, surgical burden, and genotype–phenotype correlations. Demographic (age, sex, weight, height), clinical, and laboratory data were obtained from medical records. Intestinal manifestations, including abdominal pain, alterations in stool frequency and consistency, pathological admixtures in feces, malnutrition, and perianal involvement in Crohn’s disease (CD), as well as extraintestinal manifestations (EIMs), were systematically evaluated. Laboratory markers of disease activity included complete blood count, C-reactive protein (CRP), erythrocyte sedimentation rate (ESR), protein, albumin, and fecal calprotectin. All patients underwent a comprehensive diagnostic workup, including ileocolonoscopy with systematic biopsies from all colonic segments and the terminal ileum, as well as upper gastrointestinal endoscopy with biopsies obtained irrespective of macroscopic appearance. Disease activity was assessed using the Pediatric Crohn’s Disease Activity Index (PCDAI) for CD and the Pediatric Ulcerative Colitis Activity Index (PUCAI) for ulcerative colitis (UC) and IBD-unclassified (U-IBD). Endoscopic activity was evaluated using the Simple Endoscopic Score for Crohn’s Disease (SES-CD) and the Ulcerative Colitis Endoscopic Index of Severity (UCEIS), while disease location was classified according to the Paris Classification. Histopathological assessment was conducted by experienced gastrointestinal pathologists. Histopathological evaluation included assessment of chronic inflammatory infiltrate, crypt architectural distortion, neutrophilic activity (cryptitis or crypt abscess), and the presence of granulomas. Immunologic evaluation comprised serum immunoglobulin levels (IgA, IgG, IgM, and IgE) and lymphocyte subsets (CD3^+^, CD4^+^, CD8^+^, CD19^+^, and CD56^+^ cells), which were interpreted using age-adjusted reference ranges. A family history of autoimmune, immunodeficiencies and autoinflammatory diseases, including the age at disease onset, was also documented.

### 2.2. Whole Exome Sequencing and Genetic Analysis

Genetic testing, including whole exome sequencing (WES) or whole genome sequencing (WGS), was performed in patients with clinical suspicion of monogenic disease or when permitted by resource availability. Variant interpretation followed the American College of Medical Genetics and Genomics (ACMG) guidelines. Identified variants were classified as pathogenic, likely pathogenic, variants of uncertain significance, likely benign, or benign based on population databases, in silico prediction tools, published literature, and clinical correlation. Monogenic disease was defined as the identification of pathogenic or likely pathogenic variants, classified according to ACMG criteria, in genes previously established to cause VEO-IBD or inborn errors of immunity (IEIs) with intestinal inflammation. Variants were filtered for rarity (minor allele frequency < 0.01 in population databases), predicted loss of function or deleterious missense changes, and inheritance patterns consistent with Mendelian disease models. Clinical genotype concordance was required for final classification [10].

### 2.3. Statistical Analysis

Continuous variables were assessed for normality using the Shapiro–Wilk test. Normally distributed data were presented as mean ± standard deviation, whereas non-normally distributed data were presented as median (interquartile range). Categorical variables were summarized as frequencies and percentages. Given the exploratory nature of the study and the small sample size, no formal hypothesis testing was performed. Statistical analyses were conducted using SPSS version 26.0 (IBM Corp., Armonk, NY, USA).

### 2.4. Ethical Considerations

The study was approved by the Institutional Review Board of Vietnam National Children’s Hospital (Approval No. VNCH-TRICH-678; approval date: 23 September 2025). For the prospective component, written informed consent was obtained from the parents or legal guardians of all participants prior to enrollment. For the retrospective component, the requirement for informed consent was waived by the Institutional Review Board due to the use of anonymized clinical data and minimal risk to participants.

### 2.5. Data Availability Statement

Data supporting the findings of this study are available from the corresponding author upon reasonable request. Raw genetic sequencing data are not publicly available due to institutional policies and patient confidentiality requirements. Access to anonymized data may be granted following ethical approval and institutional permission.

## 3. Results

Between July 2016 and January 2026, 36 children had symptom onset before 6 years of age and were classified as VEO-IBD, constituting the study cohort at the Vietnam National Children’s Hospital.

### 3.1. Baseline Patient Characteristic

Baseline characteristics of the VEO-IBD cohort are summarized in Table 1. A total of 36 children were included, of whom 61.1% were male. The median age at disease onset was 7.5 months (IQR 2.0–25.0), and 72.2% developed symptoms before 24 months of age. The median age at diagnosis was 12.0 months (IQR 4.0–32.0), and the median diagnostic delay was 3.0 months (IQR 1.0–4.75). Crohn’s disease was the predominant phenotype (72.2%), while ulcerative colitis and IBD-U each accounted for 13.9% of cases. A positive family history of IBD in first-degree relatives was reported in 19.4% of patients. Growth impairment, defined as HAZ < −2 and/or WAZ < −2, was observed in 75.0% of children. Stunting (HAZ < −2) was present in 61.1%, and 75.0% were underweight (WAZ < −2). WES/WGS was performed in 33 of 36 patients (91.7%). Monogenic findings were identified in 10 patients (30.3%), while 14 (42.4%) had inconclusive results. Among monogenic cases, IL10RA/IL10RB, XIAP, FOXP3, and TNFAIP3 were the most frequently identified genes (*n* = 2 each), whereas MYO5B and CYBB were detected in one patient each.

### 3.2. Clinical Phenotype and Laboratory Features

Clinical characteristics of the VEO-IBD cohort are summarized in inflammatory markers and immunologic findings are summarized in Table 2 and Supplementary Table S1. Inflammatory markers indicated substantial systemic and intestinal inflammation, with age-adjusted anemia present in 33 patients (91.7%). Median CRP, ESR, and fecal calprotectin were 25.8 mg/L (IQR 12.5–54.4), 47.0 mm/h (IQR 24.3–71.8), and 637.0 µg/g (IQR 149.3–2205.0), respectively, while median serum albumin was 32.1 g/L (IQR 29.4–36.6). Immunologic evaluation showed variable abnormalities, most notably low CD4^+^ T-cell counts in 8 patients (25.0%) and high CD8^+^ T-cell counts in 11 patients (34.4%), whereas most patients had normal CD3^+^, CD19^+^, and CD56^+^ cell counts.

Disease activity at diagnosis was substantial across the cohort. In patients with Crohn’s disease (n = 26), the median PCDAI was 38.75 (IQR 32.50–50.63), corresponding to moderate disease activity at the median (PCDAI 30–<40), with the upper IQR extending into the severe range (≥40). The median SES-CD was 18 (IQR 15–21), indicating severe endoscopic disease activity (>16). In patients with UC/IBD-U (n = 10), the median PUCAI was 47.50 (IQR 35.75–52.50), consistent with moderate disease activity (35–64), while the median UCEIS in the UC subgroup (n = 5) was 4.0 (IQR 3.0–6.0), reflecting moderate endoscopic severity. Ileocolonic disease was the predominant CD location (69.2%), and the perianal disease modifier was present in 65.4%. B1 behavior was observed in 46.2%, whereas stricturing, penetrating, and combined behavior were documented in 15.4%, 15.4%, and 26.9% of patients, respectively. Among patients with UC/IBD-U, pancolitis was the most common extent (60.0%), and severe disease (S1) was present in 30.0%. Histopathological examination most frequently showed chronic inflammatory infiltrates and mucosal ulceration (both 83.3%), followed by crypt architectural distortion (63.9%). Neutrophilic activity and granulomas were identified in 41.7% and 22.2% of patients, respectively.

Nonintestinal clinical features were common, with recurrent infections (72.2%), eczema/dermatitis (44.4%), oral ulcers (44.4%), and prolonged fever (41.7%) being the most frequent. Perianal disease affected 58.3% of patients, most commonly presenting as ulceration (44.4%), fistula (33.3%), and abscess (30.6%). Intestinal manifestations were dominated by bloody diarrhea (88.9%) and prolonged diarrhea lasting more than 14 days (86.1%), while intestinal strictures and fistulas were documented in 30.6% and 19.4% of patients, respectively.

### 3.3. Clinical Phenotypes of VEO-IBD Patients with Identified Genetic Variants

Genetic variants identified by Next Generation Sequencing (NGS) are summarized in Supplementary Table S2. The detected variants involved genes associated with epithelial barrier integrity, immune dysregulation, phagocyte dysfunction, hyperinflammatory and autoinflammatory pathways. Variants were identified in TTC7A, MYO5B, XIAP, CYBB, MEFV, MCM10, FOXP3, IL10RB, IL10RA, TNFAIP3, and IRGM. Several variants were classified as pathogenic or likely pathogenic, whereas others were categorized as variants of uncertain significance (VUS). IRGM was interpreted separately as a polygenic susceptibility finding rather than a diagnostic monogenic variant. Clinical phenotypes and treatment outcomes of patients with genetic findings are summarized in Table 3. Considerable phenotypic heterogeneity was observed; however, the dominant clinical pattern included persistent bloody or mucoid diarrhea, severe perianal disease, growth failure, recurrent infections, and extraintestinal inflammatory manifestations such as oral ulcers, eczema/dermatitis, arthritis, hepatosplenomegaly, and autoimmune features. Structural bowel damage was common and included strictures, perforation, intra-abdominal abscesses, and recurrent intestinal obstruction. Variants in IL10RA/IL10RB, XIAP, TNFAIP3, FOXP3 and MYO5B were the most frequently identified findings and were generally associated with particularly early-onset, severe, and complicated disease. Treatment burden was high, with frequent refractoriness to infliximab and adalimumab, repeated escalation to second line or adjunctive therapies, multiple surgical procedures, and HSCT in several cases.

### 3.4. Therapeutic Approaches in Children with VEO-IBD

Therapeutic approaches in children with VEO-IBD are summarized in Table 4. Nearly all patients received conventional therapy, including corticosteroids in 36 (100.0%) and both 5-ASA and immunomodulators in 35 (97.2%). Anti-TNF therapy was administered in 23 patients (63.9%), including infliximab in 19 (52.8%) and adalimumab in 8 (22.2%). Four patients (11.1%) required switching from infliximab to adalimumab due to inadequate response or loss of response. Additional targeted or adjunctive therapies included tacrolimus in 7 (19.4%), mycophenolate mofetil in 5 (13.9%), cyclosporine in 2 (5.6%), and anakinra in 1 (2.8%). IVIg was administered in 5 patients (13.9%), HSCT in 4 (11.1%), and surgical intervention was required in 15 (41.7%), including ileostomy in 11 (30.6%) and colectomy in 9 (25.0%).

## 4. Discussion

In this Vietnamese VEO-IBD cohort, Crohn’s disease was the predominant phenotype, accounting for nearly two-thirds of cases, whereas UC and U-IBD represented smaller proportions. VEO-IBD presented predominantly during infancy and was characterized by a high burden of growth and nutritional impairment [3,11]. The early age at onset (median 7.5 months), with nearly three-quarters of patients presenting before 24 months, aligns with the recognized VEO-IBD phenotype and supports early evaluation for underlying monogenic disorders or immune dysregulation [12]. The high prevalence of growth and nutritional impairment at diagnosis underscores that VEO-IBD is associated with clinically meaningful systemic consequences beyond intestinal inflammation. The marked frequency of underweight, stunting, and overall growth impairment in our cohort suggests that these effects may become evident early in the disease course, likely driven by persistent inflammation, reduced dietary intake, malabsorption, and delayed diagnosis [11,13]. East Asian data further support the concept that VEO-IBD is an early-onset, frequently CD-predominant disorder with a substantial monogenic contribution. In a Chinese cohort of 54 children younger than 6 years, Fang et al. reported that CD or CD-like disease accounted for 72.2% of cases, while monogenic IBD was identified in 16.7%, with IL10 receptor defects as the predominant genetic etiology [14]. Likewise, Usami et al. reported 54 Japanese children with VEO-IBD, with a median age at onset of 18 months, among whom 64.8% had CD-type disease and 16.7% had monogenic IBD [15]. In northern India, Poddar et al. reported an even higher monogenic rate of approximately 31–32%, with immune dysregulation disorders, including IL10 receptor mutations, representing the major etiologic group [16].

Nonintestinal clinical features were highly prevalent in our cohort, particularly recurrent infections, eczema/dermatitis, oral ulcers, and prolonged fever. In VEO-IBD, these findings are clinically relevant as they reflect a broader inflammatory or immune dysregulatory phenotype rather than disease confined to the gastrointestinal tract, consistent with prior reports demonstrating a high burden of extraintestinal and systemic manifestations in monogenic IBD [8,12]. This is especially relevant in very young children (before 24 months), in whom systemic or atypical manifestations may provide early clues to more complex underlying disease biology [2]. The intestinal phenotype was also notably severe. Perianal disease was present in more than half of the cohort and included ulceration, fistula, abscess, fissure, and skin tags, while intestinal manifestations were dominated by prolonged diarrhea and bloody diarrhea. In addition, a subset of patients had already developed stricturing or fistulizing complications, indicating that structural bowel damage may occur early in the disease course. The considerable proportion of children requiring surgical intervention further underscores the aggressive nature of disease in this population. Taken together, these findings suggest that VEO-IBD in our setting often presents with a substantial inflammatory burden and clinically significant complications early in life [6,12]. This is consistent with prior reports showing that VEO-IBD, particularly forms related to IL-10 signaling defects, is often characterized by severe bloody diarrhea, growth failure, and aggressive perianal disease with abscesses, fistulas, and fissures [17].

The inflammatory and clinical profile of our cohort suggests that VEO-IBD frequently presents with a substantial disease burden at diagnosis. The high prevalence of anemia, thrombocytosis, and hypoalbuminemia, together with elevated systemic inflammatory markers including CRP, ESR, and D-dimer and markedly increased fecal calprotectin, reflects a pronounced state of both systemic and intestinal inflammation. Conventional laboratory parameters such as CRP, ESR, platelet count, hemoglobin, and albumin are well established indicators of inflammatory activity in IBD, whereas fecal calprotectin is a sensitive, noninvasive marker of intestinal mucosal inflammation that correlates closely with endoscopic disease activity (SES-CD and UCEIS), particularly in pediatric populations [18,19,20]. The immunologic profile of our cohort demonstrated selective T-cell abnormalities, with reduced CD4^+^ and increased CD8^+^ T-cell counts in a subset of patients, while overall CD3^+^, B cell, and NK cell compartments remained largely preserved. This pattern suggests immune dysregulation rather than overt immunodeficiency and supports the concept that VEO-IBD represents a heterogeneous group of disorders characterized by impaired immune regulation. Similar alterations in T-cell subsets have been reported in monogenic VEO-IBD and may provide important clues to underlying genetic etiologies [21].

The Paris classification in our cohort indicated extensive and aggressive disease, particularly in children with Crohn’s disease. Predominant ileocolonic involvement, frequent upper gastrointestinal extension, and the high proportion of complicated behavior and/or perianal disease are consistent with previous reports showing that VEO-IBD often presents with more extensive and severe phenotypes than later onset pediatric IBD. In the UC/IBD-U subgroup, the predominance of pancolitis further supports the impression of widespread intestinal inflammation at presentation, a pattern also described in other VEO-IBD cohorts and reviews [7,11]. Histopathological abnormalities were similarly prominent, with chronic inflammatory infiltrates, mucosal ulceration, crypt architectural distortion, and granuloma formation, indicating both active inflammation and established mucosal injury at diagnosis. These features are well recognized in pediatric IBD and VEO-IBD, and the presence of granulomas together with focal crypt architectural distortion is particularly supportive of Crohn’s disease [22]. These findings suggest that VEO-IBD in our setting frequently presents with extensive intestinal involvement and early structural damage, underscoring the importance of early endoscopic assessment with systematic biopsy and comprehensive phenotypic evaluation in very young children with suspected disease [7,23].

The treatment profile of our cohort underscores the substantial severity and clinical complexity of VEO-IBD. The high frequency of surgical intervention, including ileostomy and colectomy, indicates a considerable burden of aggressive and medically refractory disease. Moreover, the use of IVIg and HSCT in selected patients supports the presence of broader immune dysregulation in a subset of cases. [24]. In our cohort, the high prevalence of ileostomy, colectomy, and growth impairment suggests a substantial burden of severe intestinal compromise and possible intestinal failure (IF)-related morbidity in a subset of patients. Nutritional management represents a cornerstone of care in children with VEO-IBD, particularly in those with severe disease and significant growth impairment. Enteral nutrition, including exclusive enteral nutrition (EEN), remains a well-established therapeutic strategy in pediatric Crohn’s disease because of its anti-inflammatory and nutritional benefits [25]. However, in patients with severe disease, extensive intestinal involvement, or postoperative complications, parenteral nutrition (PN) may be required to maintain adequate growth and nutritional status. Long-term dependence on PN is associated with important complications, including intestinal failure-associated liver disease (IFALD), catheter-related infections, and metabolic disturbances. Therefore, intestinal rehabilitation strategies aimed at promoting enteral autonomy and reducing PN dependence are important components of management [24]. Early integration of specialized nutritional support may improve growth outcomes and reduce long-term morbidity in this vulnerable population [26]. Because data on parenteral nutrition exposure, duration of intravenous support, and nutrition-related complications were not systematically collected in this retrospective cohort, these aspects could not be analyzed directly and are discussed here as important clinical considerations. Repeated surgical interventions in children with VEO-IBD may have substantial long-term consequences beyond immediate disease control. In addition to reflecting severe intestinal disease, procedures such as ileostomy and colectomy may be associated with ongoing intestinal dysfunction, impaired absorptive capacity, growth failure, and increased long-term morbidity. Children may also experience prolonged hospitalization, delayed growth and development, and reduced quality of life, while families may face substantial emotional and caregiving burdens [27,28].

Whole exome or genome sequencing was implemented in the vast majority of our cohort, underscoring the central role of genomic testing in the evaluation of children with VEO-IBD [21]. The monogenic diagnostic yield of 30.3% in our cohort is at the upper range of previously reported estimates. Prior studies suggest that approximately 13–31% of children with VEO-IBD have an identifiable monogenic etiology, with higher rates observed in cohorts enriched for infantile onset disease, severe phenotypes, or those undergoing comprehensive genomic evaluation [12]. This relatively high yield in our study likely reflects the tertiary referral setting and the high proportion of patients presenting with severe, refractory disease, complex perianal involvement, and prominent extraintestinal manifestation features that are strongly associated with underlying monogenic disorders [16,29]. Current expert guidance recommends genomic investigation as an integral component of care in children at risk for monogenic IBD because the identification of a causal variant may directly influence prognosis, genetic counseling, and treatment selection, including targeted immunomodulation or hematopoietic stem cell transplantation (HSCT) in selected disorders [12,21]. An additional notable finding was the high proportion of patients with inconclusive genetic results. This likely reflects the biological and interpretative complexity of VEO-IBD, in which variants of uncertain significance (VUS), incomplete genotype–phenotype correlation, and still undiscovered disease genes remain common challenges. Recent reviews emphasize that inconclusive findings should not be interpreted as evidence against a genetic contribution, but rather as an indication that the current diagnostic framework remains incomplete and that reanalysis over time, coupled with careful phenotyping, may improve diagnostic resolution [8].

A notable finding of our study was the high proportion of variants of uncertain significance (VUS), underscoring the persistent challenges of genetic interpretation in VEO-IBD. Although the ACMG framework provides a standardized method for variant classification, its application in rare, complex, and phenotypically heterogeneous disorders remains limited, particularly when functional evidence is lacking [10]. In this setting, genotype–phenotype correlation is critical and should be assessed in the context of established gene–disease associations, clinical concordance, and inheritance pattern, when available. Importantly, VUS should not be interpreted as definitive evidence of monogenic disease, but rather as inconclusive findings requiring cautious clinical contextualization. The absence of functional validation further restricts causal inference and increases the risk of variant misclassification, highlighting the need for integrative approaches that combine genomic, clinical, and functional data to refine diagnostic interpretation in children with VEO-IBD [6,30].

In our cohort, patient-level clinicogenetic profiling showed that children with monogenic findings generally presented with a particularly severe and syndromic phenotype, characterized by very early disease onset, recurrent infections, dermatitis/eczema, oral ulcers, growth failure, perianal disease, and complicated intestinal involvement, including strictures, perforation, and abscess formation. Many of these patients were refractory to corticosteroids and anti-TNF therapy (IFX and ADA), and several ultimately required surgical diversion or HSCT. This pattern is highly consistent with current concepts of monogenic VEO-IBD, in which intestinal inflammation often represents one manifestation of a broader disorder of immune regulation, host defense, or epithelial barrier dysfunction rather than isolated polygenic IBD [31]. Expert position papers from NASPGHAN and ESPGHAN emphasize that severe infantile onset, recurrent or unusual infections, marked perianal disease, and prominent extraintestinal or autoimmune features should raise a strong suspicion for an underlying monogenic disorder and justify comprehensive genomic evaluation [12,21].

Defects in the IL-10 signaling pathway (IL10RA/IL10RB) appear to be disproportionately more frequent in Asia, particularly in East Asian populations, whereas conditions such as IPEX (FOXP3), TTC7A deficiency, and TTC37-related disease are more commonly reported in Europe and North America [5,16,32,33]. These differences likely reflect population-specific genetic architecture and have important implications for the prioritization of genetic testing strategies. In comparison with these global patterns, our cohort demonstrates a broadly concordant distribution, with IL10RA/IL10RB, XIAP, FOXP3, and TNFAIP3 emerging as the most frequently identified genes, while MYO5B and CYBB were less commonly observed [34]. Notably, the predominance of IL-10 pathway defects and immune dysregulation-associated genes in our cohort reinforces observations from Asian populations and supports the concept that monogenic VEO-IBD in this region is enriched for defects in immune regulatory pathways [32]. A notable finding in our series was the phenotypic clustering of IL10RA/IL10RB defects with severe early-onset Crohn’s disease, especially persistent bloody diarrhea, complex perianal disease, recurrent infections, oral ulcers, growth failure, penetrating or stricturing complications, and early resistance to conventional therapy [35]. Importantly, recognition of IL-10 receptor deficiency has direct therapeutic implications because HSCT may be disease-modifying or potentially curative in appropriately selected patients [32,33,36].

The two male patients with XIAP-associated disease in our cohort also illustrate the breadth of immune dysregulatory VEO-IBD. One had severe complex perianal disease, recurrent infections, hepatomegaly, transfusion dependent anemia, and ultimately underwent bowel resection and HSCT. The other showed abdominal inflammation with hepatosplenomegaly, cytopenia, elevated liver enzymes, and a family background suggestive of hemophagocytic susceptibility. These features are concordant with the known phenotype of XIAP deficiency, which can manifest with Crohn’s-like colitis, perianal fistulizing disease, splenomegaly, hepatobiliary involvement, and HLH predisposition [37,38]. In such cases, establishing the molecular diagnosis is particularly important because therapeutic planning extends beyond standard IBD algorithms and may include transplant-based strategies [34,39]. Conventional therapy with corticosteroids and immunosuppressants resulted in limited clinical response. Anti-TNF therapy was ineffective in one patient. Additional treatment included IVIg and tacrolimus. Surgical bowel resection with diverting ileostomy was required in one case. HSCT was performed in one patient and was planned in the other, who was awaiting a suitable donor.

Similarly, the FOXP3 mutated cases in our cohort exhibited a highly syndromic phenotype, characterized by persistent bloody diarrhea, severe dermatitis, recurrent infections, oral ulcers, thyroid dysfunction, cytopenias, seizures, hypoalbuminemia, and growth impairment. This constellation strongly supports the interpretation of intestinal inflammation as part of a broader IPEX spectrum immune dysregulation, rather than isolated colitis. These findings are consistent with the established clinical spectrum of FOXP3 deficiency, in which early-onset enteropathy is typically accompanied by eczema and multi organ autoimmunity, including endocrinopathies and hematologic abnormalities. Mechanistically, FOXP3 is essential for the development and function of regulatory T cells (Treg), and its deficiency results in profound loss of immune tolerance, leading to widespread autoimmune and inflammatory manifestations [40,41]. Despite understanding IPEX pathogenesis, new treatment options have remained elusive, although early diagnosis led to HSCT and immunosuppression treatment and improved patient outcomes [42].

Defects in adaptive immune regulation were identified in patients with TNFAIP3 (A20 haploinsufficiency) variants (n = 2), exhibited syndromic features consistent with A20 haploinsufficiency (HA20), including recurrent bloody diarrhea, persistent fever, oral ulcers, arthritis, dermatitis, and growth failure, accompanied by autoimmune and immunodeficiency-like manifestations. Notably, a family history of EBV-associated hemophagocytic lymphohistiocytosis further supported an underlying immune dysregulatory disorder. This phenotype aligns with prior reports describing HA20 as an autoinflammatory condition with overlapping autoimmune features, in which gastrointestinal inflammation may mimic pediatric IBD and coexist with Behçet-like manifestations [43]. Mechanistically, TNFAIP3 encodes A20, a key negative regulator of NF-κB signaling, and its deficiency leads to dysregulated inflammatory activation across innate and adaptive immune pathways [44]. Importantly, both patients responded to Adalimumab, supporting the role of targeted biologic treatment. These findings highlight that TNFAIP3-related disease lies at the interface between autoinflammation and intestinal inflammation, and underscore the importance of molecular diagnosis in guiding pathway-based therapeutic stratification beyond conventional IBD management [45,46].

Patients with MYO5B mutations in our cohort exhibited a severe, dual phenotype encompassing both neonatal-onset enterocolitis and early-onset Crohn’s-like disease. Neonatal cases presented with intractable diarrhea, prematurity, malabsorption, and systemic complications, whereas later-onset cases developed chronic bloody diarrhea, complex perianal disease, strictures, perforation, and growth failure, often with recurrent infections. Notably, most patients showed poor response to corticosteroids and anti-TNF therapy and frequently required surgical intervention, supporting a predominantly epithelial, rather than immune-mediated, disease mechanism. These findings highlight MYO5B deficiency as a distinct epithelial driven subtype of VEO-IBD and underscore the need for alternative, mechanism-based management strategies beyond conventional immunosuppression, reinforcing the marked pathogenic heterogeneity of monogenic VEO-IBD [47,48,49,50].

The patient with CYBB-associated disease in our cohort highlights the importance of recognizing phagocyte defects in VEO-IBD, particularly when colitis coexists with recurrent bacterial infections, suppurative skin disease, or osteomyelitis suggestive of chronic granulomatous disease (CGD). This phenotype reflects impaired microbial killing due to defective NADPH oxidase activity, resulting in persistent inflammation and CGD-associated colitis that may mimic IBD. More broadly, our monogenic and genetically inconclusive cases underscore that VEO-IBD arises from diverse pathogenic mechanisms, including defective host defense, dysregulated immune signaling, and epithelial barrier dysfunction [51]. Consistent with contemporary frameworks, monogenic IBD should therefore be viewed as a mechanistically heterogeneous group of disorders converging on early-onset intestinal inflammation, with direct implications for pathway-based diagnosis and precision therapy [52]. The penetrance of CGD-associated colitis increases with age, and clinical manifestations may be subtle, necessitating a low threshold for endoscopic evaluation. Careful infection surveillance is essential during immunosuppressive therapy to mitigate the risk of opportunistic infections. Multidisciplinary management, involving close collaboration between pediatric immunologists and gastroenterologists, is critical for optimal long-term care [53].

In resource-constrained settings, the clinical application of precision medicine in VEO-IBD should rely on careful prioritization of genetic testing. Genetic evaluation is most strongly indicated in children with infantile onset, severe or refractory intestinal inflammation, recurrent infections, prominent perianal disease, growth failure, autoimmune or syndromic manifestations, or a suggestive family history. Under these conditions, a stepwise approach based on detailed phenotyping, exclusion of infection, basic immunologic work-up, and selective genetic testing may be more feasible and clinically meaningful [6,21,25].

## 5. Conclusions

This study highlights the clinical and genetic heterogeneity of VEO-IBD in a Vietnamese pediatric cohort, with a substantial proportion of patients harboring monogenic etiologies involving immune regulatory, host defense, and epithelial pathways. Monogenic disease was associated with early-onset, severe, and syndromic phenotypes, demonstrating clear genotype–phenotype correlations. These findings underscore the importance of comprehensive genetic evaluation in children with suspected VEO-IBD, particularly those with atypical or refractory disease. Early identification of underlying molecular defects enables mechanism-based therapeutic strategies, including targeted biologic therapy and hematopoietic stem cell transplantation in selected patients, supporting a shift toward precision medicine in pediatric IBD.

## Figures and Tables

**Table 1:** Baseline characteristics of children with VEO-IBD.

Variables	Total (*n* = 36)
**Demographic characteristics**	
Male sex, n (%)	22 (61.1)
Age at onset < 24 months, n (%)	26 (72.2)
Age at onset, months, median (IQR)	7.5 (2.0–25.0)
Age at diagnosis, months, median (IQR)	12.0 (4.0–32.0)
Diagnostic delay, months, median (IQR)	3.0 (1.0–4.75)
**Disease characteristics**	
Crohn’s disease, n (%)	26 (72.2)
Ulcerative colitis, n (%)	5 (13.9)
IBD-U, n (%)	5 (13.9)
Family history of IBD in a first-degree relative, n (%)	7 (19.4)
**Growth and Nutritional Status**	
Growth impairment (HAZ < −2 and/or WAZ < −2), n (%)	27 (75.0)
Stunting (HAZ < −2), n (%)	22 (61.1)
Underweight (WAZ < −2), n (%)	27 (75.0)
**Genetic evaluation (WES/WGS)**	
WES/WGS performed, n (%)	33 (91.7)
Negative, n (%)	9 (27.3)
Inconclusive findings, n (%)	14 (42.4)
Monogenic findings, n (%)	10 (30.3)
**Distribution of monogenic variants**	(n = 10)
IL10RA/IL10RB	2
XIAP	2
FOXP3	2
TNFAIP3	2
MYO5B	1
CYBB	1

CYBB: Cytochrome b-245 beta chain, FOXP3: Forkhead box P3, HAZ: Height-for-age z-score, IQR: Interquartile range, IBD-U: Inflammatory Bowel Disease Unclassified, IL10RA: Interleukin-10 receptor subunit alpha, IL10RB: Interleukin-10 receptor subunit beta, TNFAIP3: Tumor necrosis factor alpha-induced protein 3, MYO5B: Myosin VB, VEO-IBD: Very-early-onset inflammatory bowel disease, WAZ: Weight-for-age z-score, WES: Whole Exome Sequencing, WGS: Whole Genome Sequencing, XIAP: X-linked inhibitor of apoptosis protein deficiency.

**Table 2:** Clinical phenotype and laboratory features of children with VEO-IBD.

Variables	Total (*n* = 36)
**Nonintestinal clinical features**	
Recurrent infections, n (%)	26 (72.2)
Prolonged fever, n (%)	15 (41.7)
Eczema/Dermatitis n (%)	16 (44.4)
Oral ulcers, n (%)	16 (44.4)
Arthritis/Arthralgia, n (%)	8 (22.2)
Hepatobiliary involvement, n (%)	3 (8.3)
Hypothyroidism, n (%)	3 (8.3)
**Perianal disease**	21 (58.3)
Perianal ulcer, n (%)	16 (44.4)
Perianal fistulas, n (%)	12 (33.3)
Perianal abscess, n (%)	11 (30.6)
Anal fissure, n (%)	6 (16.7)
Perianal skin tags, n (%)	3 (8.3)
**Intestinal manifestations**	
Prolonged diarrhea (duration > 14 days), n (%)	31 (86.1)
Bloody diarrhea, n (%)	32 (88.9)
Intestinal stricture, n (%)	11 (30.6)
Intestinal fistula, n (%)	7 (19.4)
**Inflammatory markers**	
Age-adjusted anemia, n (%)	33 (91.7)
Hemoglobin, g/L, median (IQR)	97.5 (85.3–109.0)
Platelet count, ×10^9^/L, median (IQR)	546.5 (403.3–660.5)
D-dimer, ng/mL, median (IQR)	1101.0 (697.3–1741.0)
CRP, mg/L, median (IQR)	25.8 (12.5–54.4)
Serum Albumin, g/L, median (IQR)	32.1 (29.4–36.6)
ESR, mm/h, median (IQR)	47.0 (24.3–71.8)
Fecal calprotectin, µg/g, median (IQR)	637.0 (149.3–2205.0)
**Disease activity at diagnosis**	
PCDAI in CD (n = 26), median (IQR)	38.75 (32.50–50.63)
SES-CD in CD (n = 26), median (IQR)	18 (15–21)
PUCAI in UC/IBD-U (n = 10), median (IQR)	47.50 (35.75–52.50)
UCEIS in UC (n = 5), median (IQR)	4 (3–6)
**Paris classification (CD location)**	CD subgroup (*n* = 26)
L1: Terminal ileal ± limited cecal disease, n (%)	0
L2: Colonic, n (%)	8 (30.8)
L3: Ileocolonic, n (%)	18 (69.2)
L4a: Upper disease proximal to ligament of Treitz, n (%)	7 (26.9)
L4b: Upper disease distal to ligament of Treitz and proximal to distal 1/3 ileum, n (%)	4 (15.4)
**Paris classification (CD behavior)**	CD subgroup (*n* = 26)
B1: Non-stricturing, non-penetrating, n (%)	12 (46.2)
B2: Stricturing, n (%)	4 (15.4)
B3: Penetrating, n (%)	4 (15.4)
B2B3: Stricturing and penetrating, n (%)	7 (26.9)
Perianal disease modifier, n (%)	17 (65.4)
**Paris classification (UC/U-IBD) extent**	UC/IBD-U subgroup (*n* = 10)
E1: Ulcerative proctitis, n	1
E2: Left-sided UC, n	0
E3: Extensive UC, n	3
E4: Pancolitis, n	6
**Severity modifier (UC/U-IBD)**	UC/IBD-U subgroup (*n* = 10)
S0: Never severe, n	7
S1: Ever severe, n	3
**Histopathology**	
Chronic inflammatory infiltrate, n (%)	30 (83.3)
Crypt architectural distortion, n (%)	23 (63.9)
Neutrophilic activity (cryptitis/crypt abscess), n (%)	15 (41.7)
Granuloma, n (%)	8 (22.2)
Mucosal ulceration, n (%)	30 (83.3)

CD: Cluster of Differentiation, CRP: C-reactive protein, ESR: Erythrocyte Sedimentation Rate, IQR: Interquartile range, UC: Ulcerative colitis, U-IBD: Unclassified inflammatory bowel disease, UCEIS: Ulcerative Colitis Endoscopic Index of Severity, PCDAI: Pediatric Crohn’s Disease Activity Index, PUCAI: Pediatric Ulcerative Colitis Activity Index, SES-CD: Simple Endoscopic Score for Crohn’s Disease.

**Table 3:** Clinical Phenotypes and Genetic Findings in Patients with VEO-IBD.

ID	Sex	T1	T2	IBD	FH	Gene	Major Clinical Manifestations	Treatment and Outcome
P01/G2	F	12	12	IBDU	No	TTC7A	Recurrent intestinal obstruction, chronic diarrhea, leukopenia/neutropenia, bronchiectasis, respiratory infections, pancreatitis, severe malnutrition, and growth failure.	(TMP-SMX + Itraconazole) prophylaxis + Mesalazine + AZT + Steroid + IVIg. Poor response to IFX.
P02/G2	F	12	20	CD	No	MYO5B	Chronic bloody diarrhea, perianal disease, small bowel perforation, intestinal stricture, growth failure, persistent fever, severe eczema/dermatitis, and recurrent oral ulcers.	Steroid + IFX refractory + ADA + Ileostomy, abscess surgery, transverse colectomy.
P03/G1	M	1	1	IBDU	No	MYO5B	Neonatal onset enterocolitis, prematurity, low birth weight (35 weeks + 2100 g), chronic diarrhea, left colonic stricture, severe malnutrition, EBV infection, and fungal infection.	Prolonged neonatal mechanical ventilation. Mesalazine + AZT + Steroid. Poor response to IFX.
P04/G2	F	0.5	0.5	IBDU	Yes	MYO5B	Neonatal onset of severe enterocolitis, preterm infant (34 weeks, BW 2100 g), respiratory distress after birth, chronic muco-bloody diarrhea with malabsorption, arterial thrombosis, hypertension, neonatal jaundice.	Meropenem + Vancomycin + Fluconazole + Steroids + IVIg. Died after 2 months of treatment.
P05/G2	M	2	4	CD	No	MYO5B	Chronic bloody diarrhea, perianal disease, sigmoid stricture, Bauhin valve deformity, CMV infection.	Mesalazine + AZT + Steroid + Poor response to IFX + Ileostomy, transverse colectomy.
P06/G2	M	2	6	CD	No	MYO5B	Chronic bloody diarrhea, perianal disease, intestinal perforation with intra-abdominal abscess, sigmoid colonic stricture, and growth failure.	Mesalazine + Steroid + Poor response to IFX + Ileostomy.
P07/G1	M	6	22	CD	No	XIAP	Severe complex perianal disease, sigmoid colonic stricture, recurrent infections, prolonged fever, transfusion-dependent anemia, and hepatomegaly.	Refractory to IFX + IVIG + Tacrolimus + Bowel resection with diverting ileostomy + HSCT.
P08/G1	M	50	60	IBDU	Yes	XIAP	Abdominal pain, ileal inflammation, growth failure, hepatosplenomegaly, recurrent infections, transfusion-dependent cytopenia, elevated liver enzymes, and familial HLH susceptibility. Sibling with Crohn’s disease.	Mesalazine + UDCA + steroids + vinblastine + Repeated transfusions + Awaiting HSCT (lack of suitable donor).
P09/G1	M	10	36	IBDU	No	CYBB	Chronic bloody diarrhea, abdominal pain, persistent fever, recurrent infections (otitis media with mastoiditis, recurrent pneumonia, gingivitis, and osteomyelitis), eczema/suppurative dermatitis.	(TMP-SMX + Itraconazole) prophylaxis + Mesalazine + Steroid + Awaiting HSCT.
P10/G2	F	36	38	IBDU	Yes	MEFV	Chronic bloody diarrhea, persistent fever, elevated liver enzymes, hypercoagulability/thrombosis, polyarthritis, oral ulcers, suppurative dermatitis, malnutrition, and recurrent infectious complications.	Mesalazine + AZT, IFX + ADA refractory, Anakinra + Cyclosporine + Colchicine.
P11/G2	F	10	19	CD	No	MCM10	Chronic diarrhea, perianal ulceration and fistula, ileal stricture, Bauhin valve deformity, ankle arthritis, and eczema/dermatitis, growth impairment.	Mesalazine + AZT + steroid, Poor response to IFX + MMF. Ileostomy and abscess surgery.
P12/G1	M	1	2	IBDU	No	FOXP3	Persistent bloody diarrhea, ileocolitis, perianal ulceration, recurrent infections, severe eczema/dermatitis, oral ulcers, hypothyroidism, cholestatic liver, and hypoalbuminemia.	Mesalazine + AZT + UDCA + steroid. Refractory to IFX + Tacrolimus + IVIG + HSCT.
P13/G1	M	1.5	2	IBDU	No	FOXP3	Persistent bloody diarrhea, severe malnutrition, dermatitis, anemia, thrombocytopenia, thyroid dysfunction, hypocomplementemia, seizures, and diffuse ulcerative colitis.	Mesalazine + Steroid, + Tacrolimus + IVIG + Awaiting HSCT.
P14/G2	F	5	8	CD	No	IL10RB	Persistent bloody diarrhea, recurrent infections, severe eczema, perianal fistulizing disease, growth failure, and severe malnutrition.	Mesalazine + AZT + Refractory to steroid and IFX, Colectomy/ileostomy + Tacrolimus + Awaiting HSCT.
P15/G2	F	13	26	CD	Yes	IL10RB	Persistent mucoid bloody diarrhea, prolonged fever, oral ulcers, perianal disease, intestinal perforation with intra-abdominal abscess, growth failure, and severe malnutrition	Mesalazine + AZT + PEN + Steroid + Poor response to IFX, Ileostomy and Awaiting HSCT.
P16/G1	M	2	5	CD	No	IL10RB	Persistent mucoid bloody diarrhea, complex perianal disease, transverse colonic stricture, hepatomegaly, recurrent infections, oral ulcers, severe eczema/dermatitis, and growth failure.	Mesalazine + AZT + steroid + Refractory to IFX + IVIG + Tocilizumab + Perianal abscess surgery + HSCT.
P17/G2	M	2	6	UC	No	IL10RB	Recurrent infections, atopic dermatitis, recurrent enterocolitis episodes, chronic diarrhea, and perianal skin tags.	Mesalazine + AZT + steroid. Favorable response to IFX.
P18/G2	F	11	12	CD	No	IL10RB	Persistent mucoid bloody diarrhea, complex perianal disease (fissures, ulcers, fistula, abscess), intestinal stricture and colonic perforation, recurrent infections, oral ulcers, and growth failure.	Mesalazine + AZT + steroid, IFX refractory + ADA/Tacrolimus + colectomy with ileostomy.
P19/G2	F	2	4	CD	No	IL10RA	Persistent bloody diarrhea, recurrent infections, oral ulcers, knee arthralgia, complex perianal disease, left-sided colonic stricture, and growth failure.	Mesalazine + AZT + Steroid + PEN + Poor response to IFX and ADA, and ileostomy + Awaiting HSCT.
P20/G1	M	0.5	4	CD	Yes	IL10RA	Persistent fever, recurrent infections, severe eczema/dermatitis, oral ulcers, chronic diarrhea, perianal ulceration, hepatomegaly, and growth impairment.	Refractory to steroid and IFX + Tofacitinib, and IVIG + HSCT.
P21/G2	M	2	4	CD	No	IL10RA	Chronic diarrhea, intestinal perforation with intra-abdominal abscess, persistent fever, recurrent pneumoniae, growth impairment	Mesalazine + AZT + Ceftriaxone + Metronidazole + Poor response to IFX
P22/G1	M	48	72	CD	Yes	TNFAIP3	Abdominal pain, bloody diarrhea, perianal disease, persistent fever, recurrent oral ulcers, bilateral knee arthritis with effusion, and weight loss.	Mesalazine + AZT + Steroid. Response to ADA.
P23/G1	F	45	46	IBDU	Yes	TNFAIP3	Chronic bloody diarrhea, recurrent infections, persistent fever, dermatitis, arthritis, autoimmune thyroiditis, CID, and growth failure. Sibling died from EBV-HLH.	Mesalazine + AZT + Steroid + Response to ADA.
P24/G2	F	0.7	2	IBDU	No	IRGM	Neonatal onset inflammatory enterocolitis, persistent diarrhea, growth impairment, recurrent infections, hyperferritinemia, thrombocytosis.	Ceftriaxone + Metronidazole + Mesalazine + Steroids.

T1: Age at symptom onset (months), T2: Age at diagnosis (months), HF: Family history of IBD (first-degree relative), ADA: Adalimumab, AZT: Azathioprine, CD: Crohn’s disease, CID: Combined immunodeficiency features, F: Female, PEN: Partial enteral nutrition, HLH: Hemophagocytic lymphohistiocytosis, HSCT: Hematopoietic stem cell transplantation, G1: Monogenic findings, G2: Inconclusive findings, IBDU: Unclassified inflammatory bowel disease, IFX: Infliximab, IL10RA: Interleukin-10 receptor subunit alpha, IL10RB: Interleukin-10 receptor subunit beta, IVIg: Intravenous immunoglobulin, M: Male, MMF: Mycophenolate mofetil, TMP-SMX: Trimethoprim–sulfamethoxazole, UC: Ulcerative colitis, UDCA: Ursodeoxycholic acid, VEO-IBD: very early onset inflammatory bowel disease.

**Table 4:** Therapeutic approaches in children with VEO-IBD.

Variables	Total (*n* = 36)
**Conventional therapy**	
5-aminosalicylates (5-ASA), n (%)	35 (97.2)
Corticosteroids (oral or intravenous), n (%)	36 (100)
Immunomodulators (AZA/6MP/MTX4), n (%)	35 (97.2)
**Biologic therapy**	
Anti-TNF exposure, n (%)	23 (63.9)
Infliximab (IFX), n (%)	19 (52.8)
Adalimumab (ADA), n (%)	8 (22.2)
Anti-TNF switch (IFX → ADA), n (%)	4 (11.1)
**Targeted immune therapy**	
Mycophenolate mofetil (MMF), n (%)	5 (13.9)
Cyclosporine, n (%)	2 (5.6)
Tacrolimus, n (%)	7 (19.4)
Anakinra, n (%)	1 (2.8)
**Other therapies**	
Intravenous immunoglobulin (IVIG), n (%)	5 (13.9)
Hematopoietic Stem Cell Transplantation (HSCT), n (%)	4 (11.1)
Surgical Treatment, n (%)	15 (41.7)
Ileostomy, n (%)	11 (30.6)
Colectomy, n (%)	9 (25%)

AZA: Azathioprine, 6-MP: 6-mercaptopurine, MTX: Methotrexate.

## Data Availability

The data supporting the findings of this study are contained within the article and Supplementary Materials.

## Supplementary Materials

The following supporting information can be downloaded at: https://www.mdpi.com/article/10.3390/children13050666/s1, Table S1: Immunological Profile in Children with VEO-IBD; Table S2: Genetic Variants Identified by Next-Generation Sequencing. Table S3: STROBE Statement Checklist for “Clinical Phenotypes and Genetic Findings in Very Early Onset Inflammatory Bowel Disease: A Vietnamese Pediatric Cohort Study”.
