# Supplementary material for: Clinical Phenotypes and Genetic Findings in Very-Early-Onset Inflammatory Bowel Disease: A Vietnamese Pediatric Cohort Study
Source: Children (Basel). 2026 May 11;13(5):666. doi: 10.3390/children13050666 (PMC13204679; doi:10.3390/children13050666)
Supplement: Supplementary file 1 [file children-13-00666-s001.zip › children-4290360-supplementary.pdf]

Supplementary Materials: Immunological Profiles and Genetic Variants Identified by Next-Generation Sequencing in Children with VEO-IBD

Supplementary Table S1. Immunological Profile in Children with VEO-IBD

Variables	Total
**Humoral Immunity**	**(n = 35)**
Serum IgA, median (IQR)	1.14 (0.62–1.64)
Serum IgG, median (IQR)	10.03 (7.77–12.88)
Serum IgM, median (IQR)	1.35 (1.02–1.86)
Serum IgE, median (IQR)	194.0 (19.90–437.50)
**Cellular Immunity**	**(n = 32)**
$CD3^+$ T cells (cells/μL), median (IQR)	3904.5 (2062.0–5194.0)
$CD3^+$ low / normal / high (age-adjusted), n (%)	6 (18.8) / 20 (62.5) / 6 (18.8)
$CD4^+$ T cells (cells/μL), median (IQR)	1726.0 (1118.5–2671.8)
$CD4^+$ low / normal / high (age-adjusted), n (%)	8 (25.0) / 24 (75.0) / 0 (0.0)
$CD8^+$ T cells (cells/μL), median (IQR)	1331.0 (631.3–2061.8)
$CD8^+$ low / normal / high (age-adjusted), n (%)	5 (15.6) / 16 (50.0) / 11 (34.4)
$CD19^+$ B cells (cells/μL), median (IQR)	970.0 (463.5–1676.5)
$CD19^+$ low / normal / high (age-adjusted), n (%)	7 (21.9) / 19 (59.4) / 6 (18.8)
$CD56^+$ NK cells (cells/μL), median (IQR)	446.0 (247.3–861.2)
$CD56^+$ low / normal / high (age-adjusted), n (%)	3 (9.4) / 23 (71.9) / 6 (18.8)

Supplementary Table S2. Genetic Variants Identified by Next-Generation Sequencing

Patient ID/ Group	Gene	Variant coordinates (HGVS)	Amino acid change	Variant type	Zygosity	Inheritance	ACMG
P01/G2	TTC7A	NM_001288951.1:c.1213C>T	p.Arg405*	Nonsense	Heterozygous	AR	P
		NM_001288951.1:c.1048_1050delCTC	p.Leu350del	Inframe deletion	Heterozygous	AR	VUS
P02/G2	MYO5B	NM_001080467.2:c.1087C>T	p.Arg363*	Nonsense	Heterozygous	AR	P
		NM_001080467.2:c.1669-17G>A	p.?	Splice site	Heterozygous	AR	VUS

P03/G1	MYO5B	NM_001080467.2:c.1087C>T	p.Arg363*	Nonsense	Homozygous	AR	LP
P04/G2	MYO5B	NM_001080467.2:c.1669-17G>A; CNV del exons 3–4	p.(?)	Splice site	Heterozygous	AR	VUS
P05/G2	MYO5B	NM_001080467.3:c.947-23A>G	—	Intronic variant	Heterozygous	AR	VUS
P06/G2	MYO5B	NM_001080467.3:c.*382C>T	—	3′UTR variant	Heterozygous	AR	VUS
P07/G1	XIAP	NM_001167.4:c.776del	p.Asn259Ilefs Ter34	Frameshift	Hemizygous	XR	LP
P08/G1	XIAP	NM_001167.4:c.365del	p.Gly122Glufs Ter8	Frameshift	Hemizygous	XR	LP
P09/G1	CYBB	NM_000397.3:c.1359G>T	p.Trp453Cys	Missense	Hemizygous	XR	LP
P10/G2	MEFV	c.329T>C	p.Leu110Pro	Missense	Heterozygous	AR	LP
		c.442G>C	p.Glu148Gln	Missense	Heterozygous	AR	LP
P11* /G2	MCM10	NM_018518.5:c.2239-2A>C	Splice acceptor variant	Splice site	Heterozygous	AR	LP
		NM_018518.5:c.166G>C	p.Asp56His	Missense	Heterozygous	AR	VUS
P12/G1	FOXP3	NM_014009.4:c.1010G>A	p.Arg337Gln	Missense	Hemizygous	XR	LP
P13/G1	FOXP3	NM_014009.3:c.1190G>A	p.Arg397Gln	Missense	Hemizygous	XR	P
P14/G2	IL10RB	NM_000628.4:c.612G>C	p.Trp204Cys	Missense	Homozygous	AR	VUS
P15/G2	IL10RB	NM_000628.4:c.612G>C	p.Trp204Cys	Missense	Homozygous	AR	VUS
P16/G1	IL10RB	NM_000628.5:c.612G>C	p.Trp204Cys	Missense	Homozygous	AR	LP
P17/G2	IL10RB	NM_000628.4:c.612G>C	p.Trp204Cys	Missense	Homozygous	AR	VUS
P18/G2	IL10RB	NM_000628.4:c.612G>C	p.Trp204Cys	Missense	Homozygous	AR	VUS
P19/G2	IL10RA	NM_001558:exon6:c.784C>T	p.Arg262Cys (R262C)	Missense	Homozygous	AR	VUS
P20/G1	IL10RA	NM_001558.3:c.421G>A	p.Gly141Arg	Missense	Homozygous	AR	P
P21* /G2	IL10RA	NM_001558.4:c.421G>A	p.Gly141Arg	Missense	Heterozygous	AR	VUS

P22/G1	TNFAIP3	NM_001270507.1:c.1223C>G	p.Ser408*	Nonsense	Heterozygous	AD	P
P23/G1	TNFAIP3	NM_001270507.1:c.811C>T	p.Arg271*	Nonsense	Heterozygous	AD	P
P24*/ G2	IRGM	NM_001346557.2:c.593+1G>A	— (splice donor)	Splice site	Heterozygous	Polygenic risk locus	VUS

*ACMG: The American College of Medical Genetics and Genomics, AD: Autosomal dominant, AR: Autosomal recessive, HGVS: Human Genome Variation Society, G1: Monogenic findings, G2: Inconclusive findings LP: Likely pathogenic, P: Pathogenic, XR: X-linked recessive, UTR: Untranslated Region, VUS: Variant of uncertain significance, *: WGS: Whole Genome Sequencing.*

Table S3. STROBE Statement Checklist for "Clinical Phenotypes and Genetic Findings in Very Early Onset Inflammatory Bowel Disease: A Vietnamese Pediatric Cohort Study"

	Item No	Recommendation	Page No
**Title and abstract**	1	(*a*) Indicate the study's design with a commonly used term in the title or the abstract	1-2
		(*b*) Provide in the abstract an informative and balanced summary of what was done and what was found	1-2
**Introduction**			
Background/rationale	2	Explain the scientific background and rationale for the investigation being reported	2
Objectives	3	State specific objectives, including any prespecified hypotheses	2-3
**Methods**			
Study design	4	Present key elements of study design early in the paper	3-4
Setting	5	Describe the setting, locations, and relevant dates, including periods of recruitment, exposure, follow-up, and data collection	3-4
Participants	6	(*a*) Give the eligibility criteria, and the sources and methods of selection of participants. Describe methods of follow-up	4
		(*b*) For matched studies, give matching criteria and number of exposed and unexposed	4
Variables	7	Clearly define all outcomes, exposures, predictors, potential confounders, and effect modifiers. Give diagnostic criteria, if applicable	4

Data sources/ measurement	8*	For each variable of interest, give sources of data and details of methods of assessment (measurement). Describe comparability of assessment methods if there is more than one group	4
Bias	9	Describe any efforts to address potential sources of bias	
Study size	10	Explain how the study size was arrived at	3-5
Quantitative variables	11	Explain how quantitative variables were handled in the analyses. If applicable, describe which groupings were chosen and why	3-5
Statistical methods	12	(*a*) Describe all statistical methods, including those used to control for confounding	3-5
		(*b*) Describe any methods used to examine subgroups and interactions	3-5
		(*c*) Explain how missing data were addressed	3-5
		(*d*) If applicable, explain how loss to follow-up was addressed	3-5
		(*e*) Describe any sensitivity analyses	3-5
**Results**			
Participants	13*	(a) Report numbers of individuals at each stage of study—eg numbers potentially eligible, examined for eligibility, confirmed eligible, included in the study, completing follow-up, and analysed	5
		(b) Give reasons for non-participation at each stage	5
		(c) Consider use of a flow diagram	
Descriptive data	14*	(a) Give characteristics of study participants (eg demographic, clinical, social) and information on exposures and potential confounders	Table 1<br>Table 2
		(b) Indicate number of participants with missing data for each variable of interest	
		(c) Summarise follow-up time (eg, average and total amount)	
Outcome data	15*	Report numbers of outcome events or summary measures over time	Table 3-4<br>Table S2
Main results	16	(*a*) Give unadjusted estimates and, if applicable, confounder-adjusted estimates and their precision (eg, 95% confidence interval).	5-12

		Make clear which confounders were adjusted for and why they were included	
		(*b*) Report category boundaries when continuous variables were categorized	
		(*c*) If relevant, consider translating estimates of relative risk into absolute risk for a meaningful time period	
Other analyses	17	Report other analyses done—eg analyses of subgroups and interactions, and sensitivity analyses	
**Discussion**			
Key results	18	Summarise key results with reference to study objectives	12-17
Limitations	19	Discuss limitations of the study, taking into account sources of potential bias or imprecision. Discuss both direction and magnitude of any potential bias	12-17
Interpretation	20	Give a cautious overall interpretation of results considering objectives, limitations, multiplicity of analyses, results from similar studies, and other relevant evidence	12-17
Generalisability	21	Discuss the generalisability (external validity) of the study results	12-17
**Other information**			
Funding	22	Give the source of funding and the role of the funders for the present study and, if applicable, for the original study on which the present article is based	

*Give information separately for exposed and unexposed groups.

Note: An Explanation and Elaboration article discusses each checklist item and gives methodological background and published examples of transparent reporting. The STROBE checklist is best used in conjunction with this article (freely available on the Web sites of PLoS Medicine at http://www.plosmedicine.org/, Annals of Internal Medicine at http://www.annals.org/, and Epidemiology at http://www.epidem.com/). Information on the STROBE Initiative is available at http://www.strobe-statement.org.
